# A stepped strategy that aims at the nationwide implementation of the Enhanced Recovery After Surgery programme in major gynaecological surgery: study protocol of a cluster randomised controlled trial

**DOI:** 10.1186/s13012-015-0298-x

**Published:** 2015-07-30

**Authors:** Jeanny JA de Groot, José MC Maessen, Brigitte FM Slangen, Bjorn Winkens, Carmen D. Dirksen, Trudy van der Weijden

**Affiliations:** Department of Family Medicine, CAPHRI, School for Public Health and Primary Care, Maastricht University, P.O. Box 616, 6200 MD Maastricht, the Netherlands; Department of Obstetrics and Gynaecology, Maastricht University Medical Centre, P.O. Box 5800, 6202 AZ Maastricht, the Netherlands; Department of Quality and Safety, Maastricht University Medical Centre, P.O. Box 5800, 6202 AZ Maastricht, the Netherlands; GROW, School for Oncology and Developmental Biology, Maastricht University Medical Centre, P.O. Box 5800, 6202 AZ Maastricht, the Netherlands; Department of Methodology and Statistics, CAPHRI, School for Public Health and Primary Care, Maastricht University, P.O. Box 616, 6200 MD Maastricht, the Netherlands; Department of Clinical Epidemiology and Medical Technology Assessment, Maastricht University Medical Centre, P.O. Box 5800, 6202 AZ Maastricht, the Netherlands

**Keywords:** Comparative effectiveness research, Enhanced Recovery After Surgery, Gynaecological oncology, Large-scale implementation, Perioperative care, Quality improvement collaborative, Randomised controlled trial

## Abstract

**Background:**

Enhanced Recovery After Surgery (ERAS) programmes aim at an early recovery after surgical trauma and consequently at a reduced length of hospitalisation. This paper presents the protocol for a study that focuses on large-scale implementation of the ERAS programme in major gynaecological surgery in the Netherlands. The trial will evaluate effectiveness and costs of a stepped implementation approach that is characterised by tailoring the intensity of implementation activities to the needs of organisations and local barriers for change, in comparison with the generic breakthrough strategy that is usually applied in large-scale improvement projects in the Netherlands.

**Methods:**

All Dutch hospitals authorised to perform major abdominal surgery in gynaecological oncology patients are eligible for inclusion in this cluster randomised controlled trial. The hospitals that already fully implemented the ERAS programme in their local perioperative management or those who predominantly admit gynaecological surgery patients to an external hospital replacement care facility will be excluded. Cluster randomisation will be applied at the hospital level and will be stratified based on tertiary status. Hospitals will be randomly assigned to the stepped implementation strategy or the breakthrough strategy. The control group will receive the traditional breakthrough strategy with three educational sessions and the use of plan-do-study-act cycles for planning and executing local improvement activities. The intervention group will receive an innovative stepped strategy comprising four levels of intensity of support. Implementation starts with generic low-cost activities and may build up to the highest level of tailored and labour-intensive activities. The decision for a stepwise increase in intensive support will be based on the success of implementation so far. Both implementation strategies will be completed within 1 year and evaluated on effect, process, and cost-effectiveness. The primary outcome is length of postoperative hospital stay. Additional outcome measures are length of recovery, guideline adherence, and mean implementation costs per patient.

**Discussion:**

This study takes up the challenge to evaluate an efficient strategy for large-scale implementation. Comparing effectiveness and costs of two different approaches, this study will help to define a preferred strategy for nationwide dissemination of best practices.

**Trial registration:**

Dutch Trial Register NTR4058

## Background

Malignant neoplasms are the most important indication for major surgery in gynaecology in the Netherlands each year. Approximately 2500 women undergo gynaecological surgery for ovarian, endometrial, or cervical cancer. In the last decades, several efforts have been made in trying to reach optimal perioperative management. In the late 1990s, Kehlet introduced a renewal process aiming to maintain normal physiology after surgery [1]. Several evidence-based elements were combined in a perioperative management programme, designed to enhance recovery from major surgical trauma [2]. The Enhanced Recovery After Surgery (ERAS) programme proved to be safe with favourable effects on recovery and length of stay [3–6]. Although this programme has been successfully implemented in elective colonic surgery, a spontaneous diffusion of the ERAS programme in other elective surgery areas did not occur [7–11]. Patients undergoing major surgical procedures remain subjected to unpleasant, unnecessary, and harmful practices, despite of a large body of evidence supporting perioperative management improvements [12–14]. As a result, patients are exposed to an unnecessary prolonged hospital stay. Efficient implementation of the ERAS programme in elective gynaecological surgery on a large scale is challenging.

In 2010, the ERAS programme was successfully introduced in major gynaecological surgery at the Department of Gynaecology of Maastricht University Medical Centre [11]. This resulted in a significant reduction in length of hospital stay. Nationwide implementation of the ERAS programme in elective gynaecological surgery did not happen yet, while this is necessary to achieve high standards of care and evidence-based practice, with subsequent benefits for the successful execution of clinical trials within this field [15, 16]. Further, reduced hospitalisation will save costs and will facilitate the increased patient load by the imminent centralisation of major gynaecological surgery in the assigned high-volume centres in the Netherlands. Finally, a reduced functional decline after surgery will ease the required early start of postoperative adjuvant chemotherapy in patients with ovarian cancer.

In the Netherlands, the quality improvement model known as the Breakthrough Series [17] is currently adopted as the preferred method for health care improvement on a large scale, despite some serious drawbacks [18]. This structured collaborative learning strategy is designed to help health care organisations attain best practice in a certain field. Systematic evaluations have shown that effectiveness of the breakthrough approach varies and is at the best moderate [19]. Furthermore, breakthrough projects represent substantial investments of time, effort, and funding and are assumed to be expensive [20]. A new approach designed to deliver an optimal effect of implementation efforts at the lowest possible implementation costs is desirable. Based on the underlying assumption that organisations may vary widely in their knowledge, motivation, and capacity to run a quality improvement programme, as prescribed in the meta-evaluation of ten such programmes in the Netherlands [21], a stepped implementation strategy was designed. This strategy aims to tailor the implementation efforts to the variety in needs and local barriers to change. It offers generic low-intensive support for those who smoothly implement the changes and a more tailored and intensive approach for those hospitals having difficulties in making the changes. This differentiation of the implementation activities to preparedness for change is described in various theories on the different phases in a process of change. The Model for Planning Change of Grol describes ten stages, subdivided into awareness, understanding, acceptance, adoption, and integration of the proposed behaviour [22, 23]. This model was used to select the change interventions that will be used in the stepped implementation strategy. Each step leads to a more intensive stage in the process of change. A tailored approach is intensive and seems less suitable for large-scale improvement as it requires local support and elaborative preparatory activities [24, 25]. Evaluation of the process of this implementation effort will provide insight on why and for whom the developed strategy works and improves the understanding of the stepped approach. This will help to define a preferred strategy for nationwide dissemination of best practices.

### Objectives

The objective of this study is to compare effectiveness and costs of a stepped implementation strategy versus the generic traditional breakthrough methodology for the nationwide uptake of the ERAS approach. In addition, the current study aims to identify intervention fidelity and contextual factors that influence implementation by means of a process evaluation. The overall aim is to define a preferred strategy for the large-scale implementation of evidence-based practice. This paper describes the study protocol.

## Methods

### Design

**Fig. 1:**
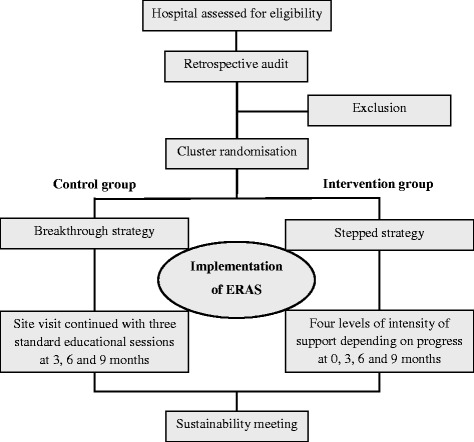
Flowchart SINERGY trial

The present study is a non-blinded cluster randomised controlled trial with two arms comparing an innovative stepwise implementation approach to the existing generic breakthrough approach. After approval to participate, a retrospective baseline measurement of 30 patients will be performed to assess current perioperative practice. Cluster randomisation will be applied at the hospital level and will take place after all retrospective measurements have been completed. Randomisation will be executed at the central level by an independent statistician of Maastricht University. The hospitals will be allocated to the intervention (stepped strategy) or to the control group (breakthrough strategy) (Fig. 1). After 1 year, the implementation process will be ended by a final sustainability meeting for both groups. We assume differences in uptake of innovations between the high-volume tertiary care hospitals and their affiliated regional hospitals. Stratified randomisation will be used to prevent unbalanced intervention groups with respect to tertiary status or not.

### Participants

#### Hospitals

All Dutch Gynaecology Oncology Group (DGOG) tertiary referral hospitals and their affiliated regional hospitals authorised to perform major gynaecological surgery will be invited to participate in this implementation study. The authorisation is based on the volume norm set by the Dutch Association of Gynaecologists in 2013. The hospitals that already fully implemented the ERAS programme in their local perioperative management are not eligible to participate. Exclusion will be based on findings of the baseline audit. To avoid interference in outcome measures, the hospitals that predominantly admit gynaecological surgery patients to an external hospital replacement care facility after discharge will be excluded as well. The DGOG, a national collaborative of professionals involved in the treatment of women with gynaecological cancers that aims to promote (inter)national research in gynaecological cancer, supports the underlying aim to reach nationwide standardisation of perioperative care consistent with the ERAS programme. This will support the recruitment of eligible hospitals in the study. In addition, we expect that cancellation of the usual 20,000 euro fee for participation to a Dutch Institute for Healthcare Improvements (CBO)-guided breakthrough project will be an important incentive towards participation. For all participating hospitals, costs of implementation are brought back to costs of joining meetings and costs of executing local activities.

#### Professionals

The innovation focuses on a professional level, directed to individual hospitals and their key target groups. Key target groups are gynaecologists and anaesthetists involved in gynaecological surgery and nurses of the gynaecology department. Gynaecological oncologists of the tertiary hospitals assist in major ovarian surgery in the regional hospitals that are authorised to perform these procedures. These oncologists are technical experts assisting in the operating room and are not involved in pre- and postoperative care. They are judged to have no influence on the implementation of the programme in the regional hospitals.

#### Patients

Women aged over 18 years and scheduled for elective abdominal surgery for diagnosis and/or treatment of (suspected) ovarian, endometrial, or cervical cancer will be included during the 1-year implementation period. No specific exclusion criteria will be applied.

### Control group

**Fig. 2:**
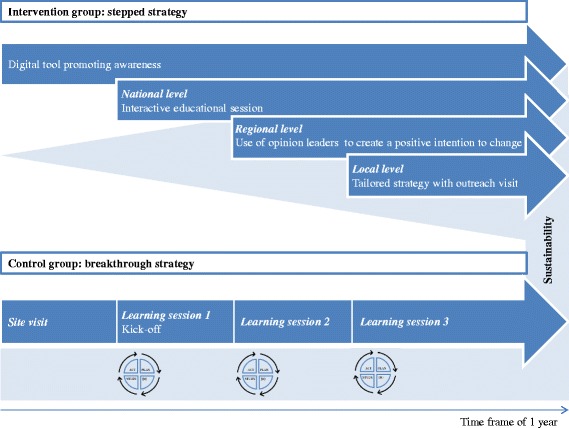
Overview of the stepped and breakthrough implementation strategy. The diagram shows the structure of the breakthrough strategy (*control group*) with standard iterative plan-do-study-act (PDSA) cycles and the stepped implementation strategy (*intervention group*) with four implementation levels depending on progress. The *light blue colour* represents the intensity of external support

Hospitals randomised to the control group will receive the traditional breakthrough strategy. The breakthrough series will be performed as usual. The CBO will chair the collaborative and recruit an expert panel including subject matter experts and an implementation expert. All participating hospitals will be instructed to appoint a multidisciplinary improvement team to lead the local improvement activities and will be visited by a CBO consultant and the researcher of the project to clarify the collaborative processes and expectations. Three educational sessions will be conducted with 3 months in between (Fig. 2). At the first learning session, the kick-off meeting, the expert team presents the vision of ideal care (the ERAS programme) and shows that actual care deviates from ideal care. The participants are taught an approach for organising and carrying out their improvement activities. This approach is called the Model for Improvement and identifies four key elements of successful process improvement: measurable aims, measures of improvement, key changes that will result in the desired performance, and a series of testing cycles, known as plan-do-study-act (PDSA) cycles [26]. This model enables teams to test change locally and then reflect, learn, and refine the changes. At the second and third learning sessions, teams learn from each other as they report on successes by feeding back their data during the learning sessions, report on lessons learned, and exchange experiences.

### Intervention group

The intervention group will be exposed to the stepped implementation strategy, which consists of four steps of implementation activities. This strategy is built on the assumptions that for some hospitals a generic improvement approach may be sufficient, while for other hospitals a full-blown breakthrough approach or an even more intensive tailored approach may be required. The four levels of intensity will be offered successively, with 3 months in between (Fig. 2).

#### First implementation level

The first implementation step aims at promoting awareness and comprises a digital toolbox available for the participating hospitals. The toolbox consists of background information of the ERAS programme, a short summary of the programme to use as a reminder in daily care, and a ready-to-use patient information sheet to structure patients’ preadmission counselling and postoperative care. We assume that this toolbox might pave the way to start implementation for those clinical leaders who have the skills to guide their department through this innovation. This assumption is fed by our experience that in the project ‘implementation of short stay in breast cancer surgery’ [27] as well as in the breakthrough project ‘perioperative care’ [28] some hospitals started immediately with treating patients according to the new programme after the programme was disseminated.

#### Second implementation level

The second step is built on support to make change happen via an interactive learning session, provided by experts in the field of implementation and experts in the implementation of the ERAS programme. The overall aim of this national meeting is to learn about the innovation and to increase motivation. The perioperative care elements and the effectiveness and safety of the ERAS programme will be discussed. Results of baseline measurement will be fed back, and the gaps between actual practice and optimal practice will be demonstrated. The subject matters of this educational meeting are comparable to the kick-off meeting in the breakthrough series, but the number of participants is limited to a maximum of three per hospital team to ensure interactive participation, promote leadership, and limit costs.

#### Third implementation level

The third step is, in contrast to the previous one, a regional activity and aims at influencing the views regarding the innovation and creating a positive intention to change. All locally involved professionals will be invited to participate in a regional meeting to learn from each other via exchange of experiences. Professional peers and opinion leaders will be used to convince them that the programme is valuable, effective, useful, and feasible.

#### Fourth implementation level

The fourth level is the most intensive one and acts on the local level. Activities comprise identification of local barriers to change and selecting activities to promote the adoption of the programme. Members of the expert team will conduct outreach visits and will guide the local team through the process of tailoring interventions. Again, audit data will be used to define to what extent the team deviates from best practice. Local cultural and environmental issues that hinder or facilitate the change process will be identified, and implementation activities will be carefully linked to these barriers and facilitators to change.

The decision to step up to a higher level of implementation activities will be based on the success of implementation. The standards of implementation success include the following: at least 80 % of patients recovered within 3 days and at least 60 % of patients going home within 5 days after surgery. Although we expect the hospitals to include about 25 patients per established evaluation period of 3 months, we assume that improvements need some time to get incorporated in routine practice. The final decision on the implementation level will be a shared decision with the hospital, which might have good reasons for clarifying the lack of success or for preferring to step up to a higher level of implementation intensity even though the benchmark of success was reached.

### Outcome measures

Process and clinical outcome data will be measured by predefined indicators of success to study contextual factors that are critical to the change process. An effectiveness, economic, and process evaluation will be conducted throughout the trial.

#### Effect evaluation

The primary outcome measure is length of hospital stay, defined as the number of nights that a patient stayed in hospital after surgery. Secondary outcome measures are length of recovery and guideline adherence. Recovery is defined as the first day after surgery that the patient fulfilled all three discharge criteria: tolerance of food, adequate pain control on oral analgesics, and independency in activities or daily living. The complete ERAS programme, as proposed by the ERAS Society® [2], includes over 20 protocol care elements. Index elements were selected as having a high level of evidence, being readily recordable, or having a direct influence on recovery. Those elements are the following: preadmission counselling, no bowel preparation, carbohydrate load, use of prophylactic antiemetics, removal of nasogastric tube on end of surgery, no peritoneal drainage, start fluids on day 0, start normal food and mobilisation on day 1, use of epidural analgesia, use of laxatives, and urinary catheter removal on day 2. Guideline adherence to the index elements will be registered for all eligible patients during the study period.

#### Economic evaluation and budget impact

An economic evaluation will be performed. Research has shown that ERAS leads to cost savings without compromising patients’ safety or quality of life [29–31]. As neither a difference in health outcomes is expected from implementation of ERAS in gynaecological surgery nor a difference in costs outside the hospital [32] (i.e. productivity costs), the economic evaluation will basically take the form of a cost-minimisation analysis and will be performed from a hospital perspective. The effectiveness of the implementation strategies (length of postoperative hospital stay) can be expressed as costs. Therefore, for both strategies, implementation costs will be calculated and compared with the cost savings due to the reduction in hospital stay. The strategy which returns the lowest net costs is considered to be the most cost-effective.

#### Process evaluation

The quantitative results will be complemented with a process evaluation. Normally, the outcomes for process evaluation are only measured in the intervention arm. In this comparative effectiveness research study, we will also measure the uptake of the implementation strategy in the control arm. The evaluation will provide valuable insights in potential barriers and facilitators that affected the effectiveness of both implementation strategies. Process data will be collected in line with a framework provided by Hulscher et al. to address actual intervention exposure and to describe experiences with the intervention [33]. Implementation fidelity will be monitored by the registration of guideline adherence to the index protocol elements during all phases of perioperative care. The registration of the number and type of professionals involved and the attendance of the educational meetings will be measured. The barriers that emerge during the educational sessions and outreach visits and the solutions to overcome these barriers will be monitored as well. Besides, all communication between the expert and local teams will be registered.

### Data collection

Before the start of implementation activities, a baseline audit of process and outcome data will be performed by reviewing medical charts of the first 30 patients treated in 2012. This period was chosen as the ERAS programme was not discussed yet in the DGOG. In case of an insufficient number of patients, the retrospective audit will be completed with patients operated in 2011. Hereafter, from the first day of the start till the end of the project 1 year later, a member of the expert team will monitor process and outcome data for both the intervention and the control group. An electronic database supporting good clinical practice guidelines will be available. Patient characteristics, process indicators, and outcome measures (proportion of patients reaching functional recovery within 3 days and proportion of patients hospitalised for a maximum of 5 days) will be audited for every 3-month period during the 1-year implementation period. The timing of the teaching sessions of the breakthrough project will be synchronous with the timing of the implementation steps (Fig. 2). Although the effectiveness of both strategies will be evaluated and compared every 3 months after every step or teaching session, the main goal of the study is to compare effectiveness at the end of the project using all patients collected during the year. Costs for the two implementation strategies will be calculated from hours and tariffs for the strategy delivery team (CBO expert, subject matter experts) as well as all costs for surgeons, anaesthetists, and nurses attending implementation-related activities, including time, travel, and conference expenses. Furthermore, the costs of materials will be calculated. Additional costs of the audit database will be calculated as well, as this is an essential part of the implementation strategies. Standardised forms will be developed for the measurement of activities, materials, and expenses associated with implementation [34]. Those forms will be filled in by members of the implementation team at each site and will provide information for the economic and process evaluation of the quality mprovement. Qualitative data regarding the process evaluation will be derived from extensive notes of all contacts with each hospital and during the educational sessions. Minutes will be taken by at least two members of the expert or local implementation team.

### Sample size

With the support of the DGOG, we expect to include the majority of Dutch hospitals and all patients that will be exposed to major gynaecological surgery during the implementation year. Approximately 100 patients undergo surgery per hospital per year. We do not expect drop outs among patients as informed consent procedures and filling in questionnaires by patients is not needed. The history of adherence to the ERAS protocol in the Netherlands during the implementation year will be described, so this study is in principle not about statistics and sample size calculations. However, it may be still worthwhile to calculate what statistical differences we will be able to assess, taking the perspective that this sample can be seen as an estimate for the future health care or assuming that some hospitals will not participate as expected. The stepped implementation strategy is expected to be superior to the breakthrough strategy in improving perioperative care processes and reducing the length of hospital stay. Anticipating a total adherence of 50 % to the benchmark of length of stay of 5 days or less in the breakthrough hospitals versus 70 % adherence in the stepped implementation hospitals, we would need to randomise seven hospitals per arm, each including at least 60 patients during the whole study period. Sample size computation was based on an estimated intra-class correlation coefficient (ICC) of 0.06 in secondary care, a power level of 80 %, and a significance level (*α*) of 0.05. The intra-class correlation coefficient used to calculate the sample size was based on a study of Campbell et al. [35]. Although this study reported significantly lower ICCs for outcome variables compared to process variables, we have conservatively computed the sample size using the highest applicable ICC of 0.06 for secondary care. Using an inflation factor of 11 % to account for unequal cluster size yields a recruitment target of 67 patients per cluster. It is possible that no clinically relevant differences (<20 %) between arms could be found. In such a case, we expect the stepped implementation programme still to be superior from a financial perspective.

### Data analysis

#### Effect evaluation

Descriptive statistics (proportions, mean, and median) will be performed for process and outcome data sets. Differences between groups will be evaluated using independent sample *t*-test for numerical variables and chi-square test for categorical variables. Since patients are nested within hospitals, implementation effects will be analysed using linear or logistic mixed-effect model analysis for numerical or binary outcome measures, accounting for a random hospital effect. Since the four implementation periods can be considered as different (time) groups, this time variable will be included as a factor in the regression model. Besides, subgroup analyses will be performed to describe trends in implementation over time in more detail. Non-inferiority comparisons will be presented for outcome measures using 95 % confidence intervals. Two-sided *p* values ≤0.05 will be considered as statistically significant. Statistical analyses will be performed using SPSS® version 22 software (SPSS, Chicago, Illinois, USA).

#### Economic evaluation and budget impact

The uncertainty as regards the cost-minimisation results will be quantified using bootstrapping techniques. Subgroup and sensitivity analysis will be performed to test for the robustness of results. An effectiveness analysis will be performed every 3 months after completing a learning session. Thus, we will be able to judge to what level of intensity the activities are worth the effort. Implementation costs and effectiveness (savings due to reduction in hospital stay) will be calculated for each period, as well as cumulative implementation costs and cumulative effectiveness. A budget impact analysis will be performed addressing the financial stream of consequences related to the uptake and diffusion of the ERAS programme to assess affordability [36]. The budget impact will depend on both the costs of both strategies and the effect in terms of hospital stay reduction as well as the current level of uptake of ERAS.

#### Process evaluation

Quantitative process data of the implementation study will be analysed by means of descriptive statistics. Potential barriers and facilitators for successful implementation of the ERAS programme will be extracted, and open text data will be categorised in codes. Content analysis will be performed to analyse the obtained qualitative data of barriers and contextual factors of the implementation process.

### Ethical approval

The Medical Ethics Committee of the University of Maastricht confirmed this quality assurance study (METC 13-5-031) was not subjected to the Dutch Clinical Research involving Human Subject Act (WMO). Therefore, informed consent at the patient level was not necessary. Privacy of patients’ information is protected by coding and processing all data anonymously.

## Trial status

Trial status at the time of manuscript submission is ongoing. The trial is currently in the last phase of implementation activities and data collection.

## Discussion

This paper presents the protocol of a cluster randomised controlled study that takes up the challenge to design an efficient strategy in large-scale implementation. Implementation of new evidence in practice is complex, and evaluations have shown a modest to moderate impact of implementation processes on the achievement of results [37]. Limited resources increase the need for more efficiency during the implementation process. Therefore, it is important to explore if there are simpler and cheaper ways to achieve the same outcomes. To our knowledge, this is the first study that used an innovative stepped strategy combining a tailored and successive implementation approach on the national level. We lack a validated instrument to measure the stage of change and the need for more or less intensive implementation strategies. In the current study, process evaluation is an important tool to gain insight why and for whom the developed implementation strategy works. Reviews of implementation strategies show that interventions are successful in some settings but fail in others [37, 38]. This stepped strategy allows us to study for whom and why pure diffusion is effective to change and for whom and why a surplus of an educational session, the use of opinion leaders, or a tailored strategy is required. Addressing the substantial gap between best practices on perioperative care and current care provided around major gynaecological surgery, the study focuses on large-scale implementation of the Enhanced Recovery After Surgery (ERAS) programme in the Netherlands. Most Dutch hospitals authorised to perform major gynaecological surgery will participate in this study, providing representative results. The ERAS programme contains several elements to promote early recovery from major surgical trauma and consequently reducing length of hospitalisation. Key index elements were selected to ensure complete and correct recording of data. Besides, data collection was carried out by a member of the expert team, limiting bias in the data collection. ERAS programmes have showed to be cost-saving [39, 30]. However, resistance to implement ERAS may arise due to competing priorities and pressure in the workplace. Those issues challenge the adoption of ERAS in both implementation groups. Process analysis will help to explore perceived barriers and facilitators and will contribute to understand and explain study outcomes. This practice-based study follows an intention to treat analysis to prevent overestimation of the implementation effect. One of the limitations of the study is the short time to evaluate the effect of the different implementations steps. Although a progressive improvement in process and clinical outcomes might be expected, the 3-month evaluation periods were chosen to follow the timing of the breakthrough educational sessions. Comparing effectiveness and costs of a stepped implementation approach with tailoring intensity of interventions to the needs of organisations to the traditionally used breakthrough strategy will help to define a preferred strategy for nationwide dissemination of best practices.

## Abbreviations

CBO: Dutch Institute for Healthcare Improvements
DGOG: Dutch Gynaecology Oncology Group
ERAS: Enhanced Recovery After Surgery
ICC: Intra-class correlation coefficient
PDSA: Plan-do-study-act
